# Anxiety among COVID-19 Patients during Their Stay in Isolation Ward in a Tertiary Care Center: A Descriptive Cross-sectional Study

**DOI:** 10.31729/jnma.7069

**Published:** 2021-10-31

**Authors:** Prabhat Rawal, Rasik Neupane, Aastha Singh, Prabina Basnet, Sudeep Chapagain, Sunder Chapagain, Rabi Paudel, Sangharsha Thapa, Rojan Pokhrel, Nirdesh Pokhrel

**Affiliations:** 1 Nepai Armed Police Force Hospital, Baiambu, Kathmandu, Nepal; 2 Kathmandu Hospital, Tripureshwor, Kathmandu, Nepal; 3 Department of Social Sciences, Tribhuvan University, Nepal; 4 Schooi of Medicine, Patan Academy of Health Sciences, Lagankhei, Laiitpur, Nepal; 5 Kathmandu University School of Medical Sciences, Dhuiikhei, Kavre, Nepal

**Keywords:** *anxiety*, *COVID-19*, *isolation*

## Abstract

**Introduction:**

Once a patient is diagnosed with COVID-19 infection, they are required to stay in isolation for some period of time without any social interaction whether be at home or in a hospital setting. The fear of contagion, stigmatization of COVID-19, the social isolation and fear of disease complication has undeniably brought lots of stress and other mental health problems among the infected patients. The main aim of this study is to find the prevalence of anxiety among patients staying in the isolation ward of a tertiary care center.

**Methods:**

This was a descriptive cross-sectional study conducted among 147 COVID-19 patients admitted in the isolation ward of a tertiary care center in Nepal from 20 June to 25 July 2021. Ethical approval was taken from Nepal Health Research Council (Reference number 3546). Convenient sampling method was used. Analysis of the data was done using Microsoft Excel and Software Statistical Package for Social Sciences version 26.0. Point estimate at 95% Confidence Interval was done and frequency and percentage were calculated.

**Results:**

Out of 147 participants, 29 (19.74.%) (95% Confidence Interval= 13.31-26.17) experienced some form of anxiety symptoms during their stay in isolation.

**Conclusions:**

Anxiety among COVID-19 patients in this study is similar to other studies done in similar settings. Anxiety among COVID-19 patients is quite high as compared to non COVID-19 patients, therefore assessment of mental health conditions like anxiety, insomnia, depression in patients admitted with COVID-19 should be performed along with the management of physical symptoms.

## INTRODUCTION

On 30^th^ January 2020, WHO declared 2019-nCoV epidemic as a Public Health Emergency of International concern.^1^ With its emergence, people are affected both physically and mentally.

Although the effects of COVID-19 are visible in physical aspects its effect on mental health is usually ignored. Once diagnosed, a patient requires staying in an isolation ward for more than two weeks without any social interaction whether be at home or in a hospital setting. The social isolation has undeniably brought lots of stress and other mental health problems among the COVID-19 patients. The immediate mental problems can be stress, anxiety and insomnia which might lead to serious mental illnesses in near future.^2^

Therefore, the main aim of this study is to find the prevalence of anxiety among patients staying in the isolation ward of a tertiary care center.

## METHODS

This study was a descriptive cross-sectional study conducted among the COVID-19 patients admitted in the isolation ward of Nepal Armed Police Force (APF) COVID-19 Hospital, Balambu, Kathmandu, Nepal conducted from 20^th^ June to 25^th^ July 2021. Ethical approval for the study was taken from Nepal Health Research Council (NHRC) (Reference no 3546). The inclusion criteria for the study were “Nepalese individuals 18 years and above admitted in the isolation ward with PCR positive COVID-19 infection in a tertiary care hospital and with a day of admission of seven or more days. Convenient sampling method was used and sample size was calculated as,

n = Z^2^ × p × q / e^2^

  = 1.96^2^ × 0.5 × 0.5 / 0.09^2^

  = 119

where,

n = required sample size,
Z = 1.96 at 95% confidence level p = prevalence taken as 50%.
q = (1-p)
e = margin of error, 9%

The calculated sample size is 119, However we took 147 participants.

A previously validated questionnaire ZUNG self-rating anxiety scale was used for the data collection. The questionnaire consisted of two sections: 1) Demographic information and 2) questions assessing anxiety level in the participants.

The participation was voluntary and written informed consent was taken from the participants willing to enroll in the study. The self-administered questionnaire was given to the participants to fill in and the data was collected through pictures of the individual form sent through the mobile application Viber. This was done to maintain the safety protocol of the institution and to prevent infection transmission from the isolation ward.

Data analysis was carried out by entering the data on Microsoft Excel and further analysis was done using Statistical Package for Social Sciences (SPSS 26.0). Point estimate at 95% Confidence Interval was done and frequency and percentage were calculated.

## RESULTS

Out of 147 participants, 29 (19.74%) (95% Confidence Interval = 13.31-26.17) experienced some form of anxiety symptoms during their stay in isolation. Among them, 26 (17.7%) had mild to moderate levels of anxiety and few 3 (2.04%) experienced marked to severe levels of anxiety. While assessing the anxiety level of the participants, the mean score was 35.28 ± 8.99 (Table 1).

**Table 1:** ZUNG Anxiety Scale interpretation.

Anxiety level	n (%)
Normal Range (20-44)	118 (80.27)
Mild to Moderate Anxiety level (45-59)	26 (17.70)
Marked to Severe Anxiety level (60­74)	3 (2.04)
Extreme Anxiety level (>75)	0 (0)

The study included 147 participants; out of which more than half were male 80 (54.4%) and others were female 67 (45.6%). Among the total participants, 49 (33.33%) of them were of age group 18-36 years followed by 56 (38.1 %) were of age group 36-54 years and 32 (21.76%) of participants were of 54-72 years. Whereas, only 10 (6.80%) of the respondents were of age 72 years and above. Of the total participants, 126 (85.7%) were married and only 21 (14.3%) were single. More than half of the respondents were unemployed 82 (55.8%) and nearly half of them were employed 65 (44.2%) which is presented in (Table 2).

**Table 2:** Demographic Information of the respondents.

Characteristics	n (%)
**Age**	
18-36	49 (33.33)
36-54	56 (38.10)
54-72	32(21.76)
Above 72	10 (6.80)
**Sex**	
Male	80 (54.40)
Female	67 (45.60)
**Occupation status**	
Employed	65 (44.20)
Unemployed	82 (55.80)
**Marital Status**	
Married	126 (85.70)
Single	21 (14.30)

While taking the medical history of each participant, nearly two third had no any chronic illnesses 101 (68.7%) while remaining of them had some chronic illnesses 46 (31.31%). Among them only few of them had some form of mental illness 7 (4.8%).

## DISCUSSION

Since the start of the pandemic, the majority of effort and resources are utilized to contain the virus. When a person contracts the coronavirus, the focus is given to treat the physical symptoms only with assessment of psychological status being ignored, although this aspect is equally important. With the spread of the COVID-19 worldwide, due to the stringent measures to contain the virus like isolation and quarantine people are experiencing various psychological problems including anxiety, depression and stress.^3^ Previously done study after the outbreak of Severe Acute Respiratory Syndrome (SARS) in 2003 and Ebola outbreak in 2014 also showed various psychological issues among the quarantined individuals with reported long term behavior changes.^4^ Although this is a very important issue that is needed to be addressed, there are very few studies done in Nepal to explore the issues related to psychological issues in real patients suffering from COVID-19. So, this study aims to determine the prevalence and severity of anxiety among the patients in a tertiary care center for isolation. Out of the 147 participants in the study, the majority of them 118 (80.27%) had normal levels of anxiety whereas none of the participants had extreme levels of anxiety. Out of 147 participants, 29 (19.74.%) (95% Confidence Interval= 13.31-26.17) experienced some form of anxiety symptoms during their stay in isolation.

In a study done in Saudi Arabia in 400 university students, 21.5% (86) participants exhibited minimal to moderate anxiety level which was comparable to our study whereas 8.8% (35) and 4.3% (17) participants showed marked to severe anxiety and extreme anxiety respectively.^5^ The high level of extreme anxiety might be due to the higher number of participants than our study. In-addition stress due to the concerns regarding the future and academic pressure along with additional mental burden due to the COVID-19 could be the reason for these findings.

In another similar study done in COVID-19 dedicated hospital in Delhi, India among 122 patients who were government employees, only 5 patients (4.4%) had moderate to severe anxiety. Remaining 117 patients in the study had scores within normal anxiety level. The lower level of anxiety might be related to the higher level of education and better care from the health care facility.^6^

In a similar study conducted in a hemodialysis center from Second People's Hospital of Yibin, China in 321 patients undergoing maintenance hemodialysis (MHD), there were 112 cases (34.89%) of MHD patients with anxiety. The number of patients with mild to moderate, moderate to severe, and extreme severe anxiety were 79 (70.53%), 30 (26.97%), 3 (2.78%) respectively. Although the patients were not suffering from the COVID-19, the finding was comparable to our study. This might be due to the prevalent chronic kidney disease among the participants. Other risk factor might be the fear of contracting the COVID-19 infection due to the regular visit for the hemodialysis.^7^

In a study done in 89 patients with confirmed COVID-19 virus in Egypt, out of the hospitalized patients 39.1% had abnormal scores of HADS-Anxiety scores which denoted considerable symptoms of anxiety which was comparable to our study.^8^

Our study reveals some form of anxiety in about 20% of the patients admitted to the isolation ward of a COVID-19 hospital during the period of our study. It signifies that not only the physical symptoms but patients are also affected with mental problems like anxiety. This might be due to the fear of consequences of the disease and social isolation. This provides us a platform to further explore such mental issues in patients during isolation not only in the hospital but also in household settings. Furthermore, public awareness regarding the psychological issues like stress, anxiety, depression and insomnia would help them to open up and seek help upon facing these problems.

## CONCLUSIONS

Anxiety among COVID-19 patients this study is similar to other studies done in similar settings. Anxiety among COVID-19 patients is quite high as compared to non COVID-19 patients, therefore assessment of mental health conditions like anxiety, insomnia, depression in patients admitted with COVID-19 should be performed along with the management of physical symptoms. The study shows that during the management of patients with COVID-19 the only focus may not be narrowed to the physical symptoms. Assessment for any form of mental health problems should also be conducted whenever a patient is admitted in a hospital setting. Timely assessment and intervention of any mental health issues like anxiety, depression, insomnia, might help to improve the quality of life and prevent any possible psychological illnesses in the near future.
